# Conductive Hearing Loss with Age—A Histologic and Audiometric Evaluation

**DOI:** 10.3390/jcm10112341

**Published:** 2021-05-27

**Authors:** Ivo Dobrev, Daniel Dillinger, Letizia Meier, Dorothe Veraguth, Flurin Pfiffner, Rudolf Probst, Christof Röösli

**Affiliations:** Department of Otorhinolaryngology, Head & Neck Surgery, University Hospital Zurich, University of Zurich, 8091 Zurich, Switzerland; ivo.dobrev@usz.ch (I.D.); daniel.dillinger@hotmail.com (D.D.); letizia.meier@luks.ch (L.M.); dorothe.veraguth@usz.ch (D.V.); flurin.pfiffner@usz.ch (F.P.); rudolfr.probst@uzh.ch (R.P.)

**Keywords:** middle ear impecance, histopathology, incudo-mallear, incudo-stapedial joint, age, air-bone gap

## Abstract

A retrospective analysis to quantify age-related changes of the incudo-malleolar joint (IMJ) and incudo-stapedial joint (ISJ), and to analyse changes in the air-bone gap (ABG) with age, was performed. Defined histologic parameters of 153 IMJ and 106 ISJ from subjects aged from birth to 70 years were correlated to age. Additionally, audiograms of 1760 ears of 974 other subjects aged 20 to 80 years were retrospectively analysed and the ABG was correlated to age. The joint space (age group from 0 to 10 compared to 61 to 70 years) became significantly wider with age (IMJ: from a mean of 44 µm to 100 µm, *p* < 0.001; ISJ: from a mean of 28 µm to 69 µm, *p* < 0.009. The thickness of cartilage of the incus decreased in the first 20 years of life (IMJ, from a mean of 88 µm to 65 µm, *p* < 0.01; ISJ: from a mean of 44 µm to 35 µm, *p* < 0.01). The ABGs of younger ears (20–40 years) was significantly larger at 500 Hz compared to older ears (60–80 years) by 2–4 dB, while it was significantly smaller by 3–5 dB at 4000 Hz (*p* < 0.0017). Interindividual variations in all age groups were large for both analyses. The increased joint spaces could potentially reduce the stiffness in the joints and explain the increase in ABG at 4000 Hz and the drop at 500 Hz. While the average change is small and of minimal clinical relevance, a larger increase of ABG with age is seen in some subjects.

## 1. Introduction

The percentage of adults with hearing loss increases with age from 5.5% in adults aged 18 to 39 years to 43% of adults 70 years or older [1]. The aging process affects primarily the cochlea and includes degeneration of auditory hair cells, cochlear neurons, atrophy of the stria vascularis, and degeneration of the spiral ligament [2]. Changes in signal processing in the central auditory systems contribute to the hearing deterioration [3]. Also an age-dependent transmission component is described. Two descriptive studies show an age-dependent air-bone gap (ABG) at 4000 Hz in males aged 25 to 80 years without noise exposure or otologic disease [4,5]. This was confirmed by Nondahl [6] with 2000 participants showing an increasing ABG with increasing age. An experimental analysis in human temporal bones (TBs) showed a trend of decreased sound transmission in the middle ear at higher frequencies (>3 kHz) with age [7]. However, others could not confirm these findings [8,9,10,11,12]. 

From a mechanical point of view, the relative motion between malleus and incus increases with frequency in the physiological condition. The relative motion of the incudo-malleolar joint (IMJ) is larger at higher frequencies and sound energy is lost. At low frequencies, there is only little relative motion between malleus and incus. Stiffening in the middle ear joints results in better sound transmission at high frequencies [13,14]. Widening the joint space of the IMJ, with corresponding softening, should result in the opposite effect, namely, worse sound transmission at high frequencies. Therefore, the aforementioned decreased sound transmission at high frequencies could be caused by structural changes of the middle ear structures.

Two anatomical structures are possible candidates for these structural changes; the tympanic membrane and the middle ear ossicles. Structural changes of the tympanic membrane during life include decreased vascularization, decreased cellularity, increased rigidity [15], reduction of elastic tissue in the tympanic cavity [16], replacement of bone structure with fibrous tissue, an increasing number of fat cells in the bone marrow in the auditory ossicles, calcification of chondrocytes of the cartilaginous part of the Eustachian tube, an increased amount of endomysium and perimysium in the stapedius muscle, and an increased quantity of fatty tissue in the tensor tympani [17]. Attempts to quantify these structural changes by using tympanometric measurements showed contradictory findings [18,19]. Additionally, single frequency tympanometry using 226 Hz showed no significant age effect when comparing young adults (18 to 28 years) to older adults (60 to 85 years) [20], indicating that tympanometry is not the optimal method to evaluate middle ear transfer function. The middle ear ossicles have been investigated histopathologically with analyses of the IMJ and the incudo-stapedial joint (ISJ). Etholm and Belal analyzed 125 normal ears of 86 individuals varying in age from infancy to 96 years [18]. They found various changes of increasing severity in the IMJ and ISJ with advancing age including degenerative modifications of the articular cartilage containing fraying and fibrillation, followed by thinning and calcification. Early changes of the articular disc consisted of hyaline deposits followed by calcium deposits with advancing age. The joint space diminished as age progressed. Gussen found in a study of 152 TBs from 90 autopsies (age 0 to 88 years) focal surface defects of the IMJ, loss of joint surface demarcation, approximation of the joint surfaces, and absent articular discs with advancing age [21]. Similar degenerative modifications of the IMJ such as atrophy, fibrosis, hyalinization, and calcification were reported by Savic and Djeric in their histological study of 40 normal TBs from individuals aged 40 to 60 years [22]. 

The aim of our study was to add more data to these conflicting findings. In particular, we aimed to (1) investigate and quantify the effect of aging on the structures of the two middle ear joints in TB specimens, and (2) analyze a large dataset of audiograms with regard to the presence of or change in an ABG with increasing age.

It is important to understand the age effects in the middle ear and their effect on the input into the cochlea to understand the aging process in the auditory system, presbyacusis, for the interpretation of otoacoustic emissions with forward and reverse transfer of sound energy in the middle ear, and to develop clinical norms for hearing and middle-ear assessment. 

## 2. Materials and Methods

### 2.1. Histological Analysis of Middle-Ear Joints

This study was approved by the Ethical Committee (KEK-Nr. 2015-0474). The TBs included in the study originated from the Department’s TB collection. They were processed by the standard method of fixation in formalin, decalcification using trichloroacetic acid or ethylenediaminetetraacetic acid, embedding in celloidin, serial sectioning at a section thickness of 20 µm, and staining of every 10th section, usually with hematoxylin and eosin [23]. The TB sections were stored in cardboard folders in a climate-controlled room.

**Figure 1:**
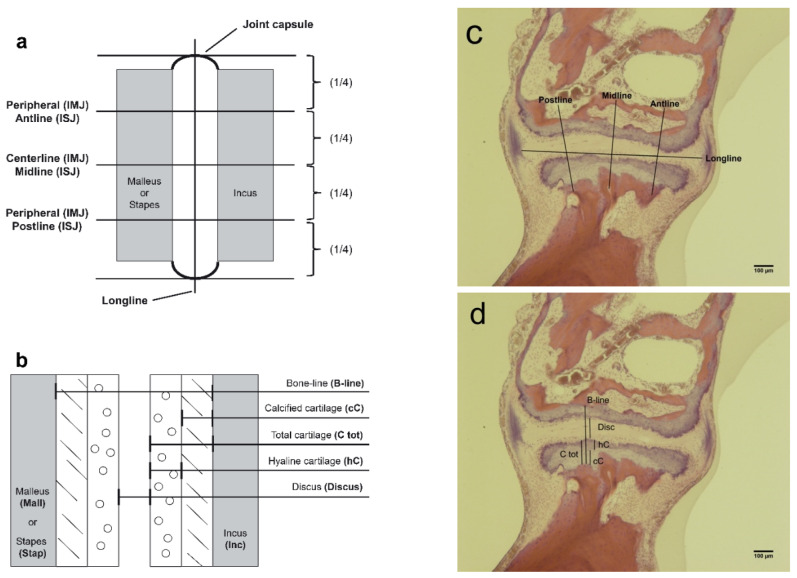
Graphical definition of the geometrical parameters defining the midsection of the incudo-malleal joint (IMJ) and the incudo-stapedial joint (ISJ), including lateral (**a**) and transverse (**b**) measurement positions for each joint (Fausch et al. [24]). Illustration of the parameters longline, midline, antline, and postline midsection of one representative incudostapedial joint (**c**), and B-line cC, C tot, hC and Discus (**d**).

Inclusion criteria were: (1) subjects aged from birth to 70 years, (2) absence of any known pathology of the middle ear, and (3) sufficient quality of TB sections without post-mortem artefacts. To identify the best histologic section of the IMJ or ISJ, all available slides from included TBs were examined using a binocular microscope (Leica DMRB; Leica Microsystems, Wetzlar, Germany). If both TBs were of equal quality, just one side (right side) was further investigated to prevent selection bias. The most representative section of each joint was digitally photographed with a resolution of 2452 × 2056 pixels using a camera (AxioCam ICc 5; Carl Zeiss AG, Oberkochen, Germany) attached to the microscope with a C-Mount optical interface (Leica Wild 543,345; Leica Microsystems, Wetzlar, Germany). Photographic magnification was 1.6× and 5.0×. Measurements were made on digital images with ImageJ (15 November 2020 https://imagej.nih.gov/ij/). For the IMJ, the measuring method has been previously described [24]. Twenty-five parameters were determined on each IMJ histologic section. The distance between the synovial membranes on the medial ligaments of the capsule in the joint line was measured and labelled as “Longline.” This line was used as reference and not for further analysis. The Longline was quartered by setting three points at which orthogonal measurement lines were positioned (Centerline and two Peripheral), as illustrated in Figure 1a. 

Several joint-thickness parameters were measured along these lines for the IMJ and ISJ, as illustrated in Figure 1b:

(1) **B-line** (Bone-line)—distance between the osseous portions of the corresponding ossicles.
(2) **Discus**—distance between the ossicles.
(3) **C tot**—total cartilage of the ossicle.
(4) **cC**—calcified cartilage of the ossicle.
(5) **hC**—hyaline cartilage of the ossicle, equal to **C tot** minus **cC.**

The total (C tot), calcified (cC), and hyaline (hC) cartilage metrics were estimated for each ossicle (Mall for malleus, Inc for incus, and Stap for stapes) in each joint (IMJ and ISJ). For example, Mall hC corresponds to the hyaline cartilage thickness of the malleus. A total of 8 parameters were measured for each quarter line.

For the IMJ, the values of the two peripheral positions were averaged because the orientation of the joint varied somewhat between sections. A total of 17 parameters (8 parameters for the central quarter line, 8 parameters for the averaged peripheral quarter line, and 1 Longline) were available for analysis from each IMJ.

For the ISJ, the same measuring method was applied resulting in a Longline and 8 parameters per quarter line for a total of 25 parameters per joint section, as illustrated in Figure 1. In contrast to the IMJ, the three quarter lines were labelled as Midline, Antline and Postline (defined as the quarter line on the end of the insertion of the stapedius muscle). The results of the measurements of the Antline and Postline were not averaged. 

This measurement approach shows high reliability, as a comparison between measurements of two independent investigators did not differ significantly in a previous study [25]. The anatomical parameters for IMJ and ISJ were correlated to the ages of the subjects.

### 2.2. Air-Bone Gap (ABG) Data

Our audiometric database of approximately 200,000 tone audiograms from 170,000 patients was searched to meet the following inclusion criteria: (1) age between 20 and 80 years; (2) age- and gender-matched normal air conduction (AC) thresholds in at least one ear for 500 Hz, 1000 Hz, 2000 Hz, and 4000 Hz according to the adapted ISO-standards (75th percentile) [26]; (3) bilateral AC hearing thresholds of 40 dB HL or better, or single-sided deafness (SSD) defined as masked bone conduction (BC) thresholds of 60 dB HL or more in one ear and AC hearing thresholds of 40 dB HL or better in the other ear; (4) no history of ear surgery; (5) when multiple audiograms were available per subject, only the most recent audiogram was used for analysis. Data from a total of 974 patients and 1760 audiograms were further analyzed, composed of 1572 audiograms of 786 normal hearing subjects, and 188 audiograms of SSD patients (116 from the right and 72 from the left ear). The demographic distribution of the patients is shown in Table 1. The ABGs of the frequencies 500 Hz, 1000 Hz, 2000 Hz, 4000 Hz, and the pure tone average (PTA) (500–4000 Hz) were analyzed for statistically significant changes with age.

### 2.3. Statistical Analysis 

For statistical analysis of the histological data, an RStudio software package (Version 0.99.896; RStudio, Inc., Boston, MA, USA) was used. Generalized cross-validation smoothness estimation was performed for all anatomical parameters (graphically defined in Figure 1) with the R-package “mgcv” to determine any age-related changes. Dependence of each parameter on age was tested for smooth terms based on Nychka’s analysis of the frequency properties of Bayesian confidence intervals for smooths [27]. A *p*-value of <0.01 was considered statistically significant. 

For processing and statistical analysis of the audiogram data, a custom Matlab script (2020a, MathWorks, MA, USA) was used. Age dependence, calculated at each frequency and PTA, was defined as a statistically significant difference in the ABG between two age groups separated by a 20-year age span: (1) the “younger” group of 20 to 40 years of age, and (2) the “older” group of 60 to 80 years of age. Statistical analysis of the difference between the two groups was done via a two-sided Wilcoxon rank sum test (function “ranksum,” equivalent to the Mann–Whitney U-test). The test was applied individually for data at all four test frequencies plus the PTA, resulting in five tests. Thus, the threshold for statistical significance was adjusted by the number of tests using the Bonferroni correction, resulting in a *p*-value of <0.0017 for analysis of the audiogram data.

## 3. Results

### 3.1. Histological Analysis of Middle-Ear Joints

**Figure 2:**
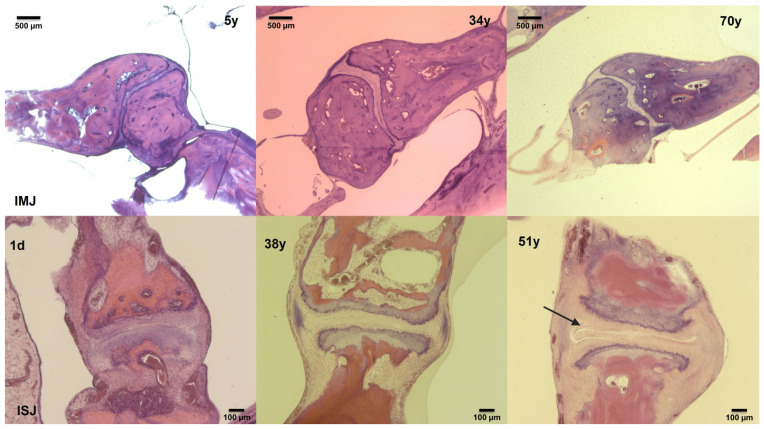
Comparison of hematoxylin- and eosin-stained histologic sections of the incudo-mallear joint (IMJ; top line) and incudo-stapedial joint (ISJ; lower line) from young (**left**), middle and older (**right**) subjects showing increasing width of the discus of the IMJ and the posterior part of the ISJ (arrow) with age. The specific age in days (d) or years (y) of each subject is displayed in each picture. All IMJ sections are presented in 1.6× magnification; the ISJ sections are in 5× magnification.

The histologic sections of 153 IMJ (102 right, 51 left) and 106 ISJ (69 right, 37 left) were analysed. Supplemental Table S1 lists the specific age, gender and side distribution for each section. An exemplary overview of the slides chosen for analysis is shown in Figure 2 for the IMJ on the top row and for the ISJ on the bottom row. In general, the variability among patients of similar age was large. The following statistically significant differences were identified.

**Figure 3:**
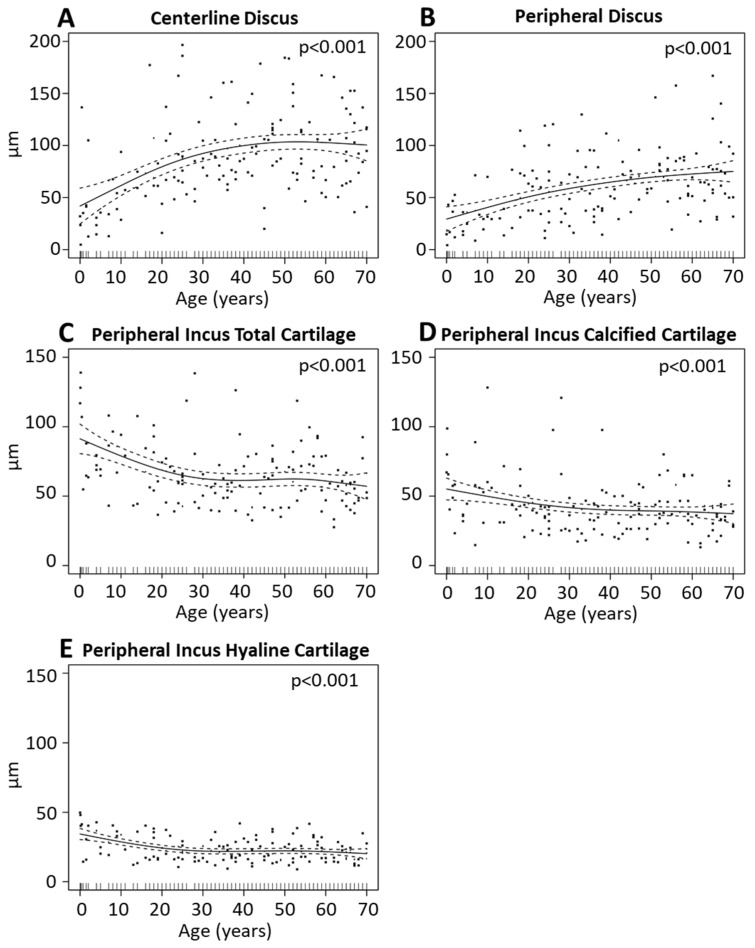
Incudo-mallear joint (IMJ) geometrical variation with age. Indicated are generalized cross-validation optimal non-parametric regression curves (solid) of the significant parameters: (**A**) Centerline Discus, (**B**) Peripheral Discus, (**C**) Peripheral Inc C tot, (**D**) Peripheral Inc cC, and (**E**) Peripheral Inc hC. The dotted lines form the 95% pointwise confidence intervals. The black dots represent single measurements. The black lines on the x-axis indicate the age of each individual.

The joint space between the incus and the malleus at the IMJ tended to get wider with advancing age (Table 2 and Supplemental Table S2; Figure 3A,B). At the central position of the joint, the joint space (Centerline Discus) widened significantly (*p* < 0.001). For the age group from 0 to 10 years, the joint space was 44 µm (range 5–136 µm) compared to 100 µm (range 36–166) for 61 to 70 years. It remained approximately (within 10%) constant from 50 to 80 years of age (Figure 3A). At the Peripheral Discus, the joint space showed a steadier widening (*p* < 0.001) of approximately 35 µm from 30 µm (range 4–71 µm) for ages 0 to 10 years to 77 µm (range 31–103 µm) for 61 to 70 years (Figure 3B). Additionally, the thickness of the cartilage at the periphery of the incus (Peripheral Inc C tot) decreased significantly (*p* < 0.001) in the first 20 years of life from a mean of 88 µm (range 43–128 µm) for ages 0 to 10 years to 65 µm (range 43–100 µm) for 21 to 30 years. Thereafter, it remained approximately (within 10%) constant (Figure 3C). The calcified cartilage at this position decreased approximately 10 µm (20%) in thickness (Peripheral Inc cC; Figure 3D; *p* = 0.006) from a mean of 55 µm (range 15–128 µm) for ages 0 to 10 years to 42 µm (range 22–120 µm) for 21 to 30 years. The hyaline cartilage of the incus (Peripheral Inc hC) also decreased to about 10 µm from a mean of 33 µm (range 14–48 µm) for ages 0 to 10 years to 22 µm (range 14–22 µm) for 21 to 30 years, corresponding to a change of 40% (Figure 3E; *p* < 0.001). All other parameters of the IMJ did not show statistically significant age-related changes, as shown in Supplemental Table S2. 

**Figure 4:**
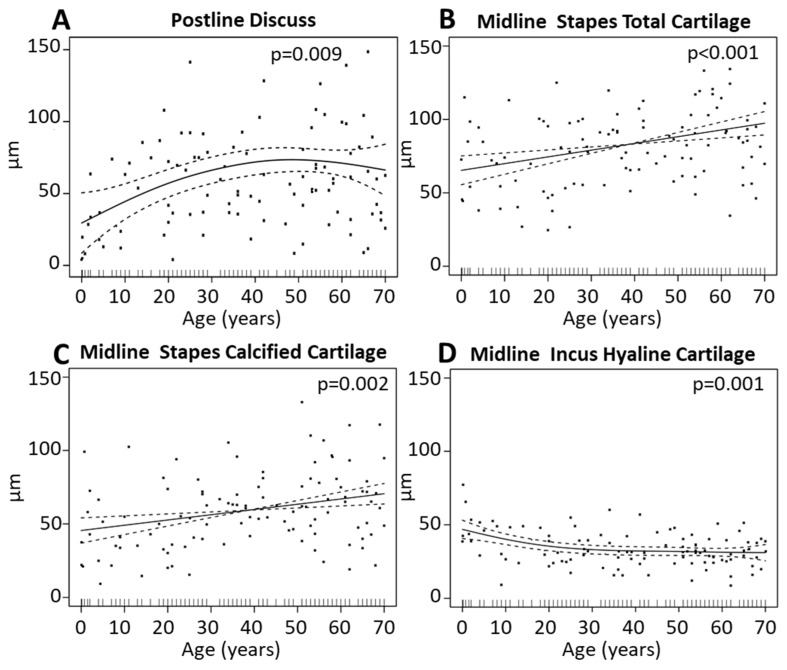
Incudo-stapedial joint (ISJ) geometrical variation with age. Indicated are generalized cross-validation optimal non-parametric regression curves (solid) of the significant parameters: (**A**) Postline Discus, (**B**) Midline Stap C tot, (**C**) Midline Stap cC, and (**D**) Midline Inc hC. The dotted lines form the 95% pointwise confidence intervals. The black dots represent single measurements. The black lines on the x-axis indicate the age of each individual.

The analysis of the ISJ showed inconsistent widening of the joint space (Table 2, Supplemental Table S3, Figure 4A). A significant (*p* = 0.009) widening of 40 µm (150%) of the joint space between the stapes and incus was seen in the posterior area of the joint (Postline Discus) from a mean of 28 µm (range 4–63 µm) to a mean of 69 µm (range 9–175 µm), *p* < 0.009. The changes after the age of 50 years of age are small and within 15% (Figure 4A). No significant change of the ISJ at the Midline or Antline was detected. The total cartilage of the stapes in the center of the joint (Midline Stap C tot) became significantly thicker (30 µm or 45% change) with age (*p* < 0.001, Figure 4B). It increased from a mean of 69 µm (range 38–98 µm) in the age group of 0 to 10 years to a mean of 101 µm (range 42–153 µm) in the age group of 61 to 70 years. The calcified cartilage of the stapes (Midline Stap cC) in the center of the joint increased by 20 µm or 65% (*p* < 0.002, Figure 4C) from a mean of 45 µm (range 21–99 µm) in the age group of 0 to 10 years to a mean of 64 µm (range 19–121 µm) in the age group of 61 to 70 years. The thickness of the hyaline cartilage of the incus in the center of the joint (Midline Inch C) showed a significant age-related decrease (10 µm or 25% change) from 44 µm (range 9–77 µm) for the age group of 0 to 10 years to 35 µm (range 17–49 µm) for the age group of 21 to 30 years (*p* = 0.001). Then it remained approximately (within 5%) constant (Figure 4D). All other parameters of the ISJ did not show statistically significant age-related changes, as shown in Supplemental Table S3.

### 3.2. ABG Patient Data

**Figure 5:**
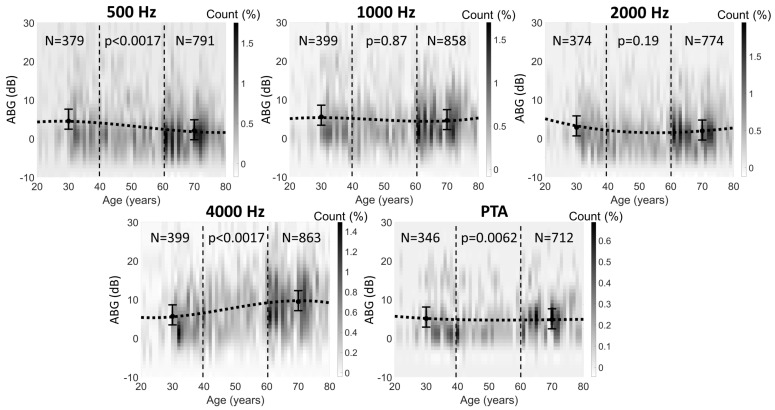
Air-bone gap (ABG) distribution with age and frequency. Error bars indicate the mean ABG and 99% confidence interval for two age groups: young (20–40 years) and old (60–80 years). The colours indicate the two-dimensional histogram (1-year and 1-dB-ABG bin size) of the data, relative to both ABG and age. Colour bars indicate the frequency of occurrence in units of percent relative to the total number of tested ears. The dotted lines correspond to a 3rd-degree spline fitted to the data point cloud, used only for illustrating the trend in the data. Vertical dashed lines provide a visual separation of the data corresponding to the younger and older age groups.

The ABGs (500, 1000, 2000, 4000 Hz, PTA) are shown in Figure 5 with the largest variability in the youngest age group. On average, the mean ABG (PTA) ranged between 4 and 7 dB for all age groups. No significant age-related change in the mean ABGs could be observed. The variability between subjects within the same age group was large.

The ABG distribution with age for individual frequencies exhibited similar findings at 1000 and 2000 Hz to those of the ABG for the PTA. Namely, no statistically significant changes in the ABGs between the two age groups (“young” versus “old”) were found for those individual frequencies. The ABG data average (indicated by a fitted 3rd-degree spline in Figure 5) at those frequencies varied between 1 and 7 dB across the age range.

At 500 Hz, the older group of subjects (60–80 years) showed significantly smaller ABGs (*p* < 0.0017; 95% CI is 2–4 dB, mean 3 dB) than the younger group (20–40 years). The trend for decreasing ABGs with age at 500 Hz was steady across the full age range. At 4000 Hz, the older group of subjects (60–80 years) showed significantly larger ABGs (*p* < 0.0017; 95% confidence interval (CI) is 3–5 dB, mean 4 dB) than the younger group (20–40 years). The trend for increasing ABG with age at 4000 Hz started at the age of 30 and continued to 60 years before levelling.

## 4. Discussion

The histologic analysis revealed two findings: an age-related increase in the joint spaces of the IMJ, and a decrease in the cartilage of the incus at both joints in the first 20 years of life.

A general significant age-related widening of the joint spaces was observed for the IMJ until the age of 50 at the central position, and until the age of 70 at the peripheral position. For the ISJ at the posterior position, the joint space widened significantly while insignificant change was observed at the centre and anterior position of the joint. These findings are in contrast to others describing a narrowing of the joint space and approximation of the joint surfaces with advancing age [18,21]. These authors either used an arbitrary scale with three degrees of severity (Grade I, mild; II, moderate; III, severe) for grading the changes of the capsule, articular cartilage, disc, and joint space in both joints [18], or the results seemed to be based on observations only with no detailed method described [21]. These descriptive approaches in comparison to our quantitative evaluations might account for our different findings. 

The audiometric measurements showed small (2–5 dB) but statistically significant (*p* < 0.0017) age-related changes in the ABGs in two frequencies: a decrease of the ABG at 500 Hz and an increase at 4000 Hz. Even though the audiometric changes are statistically significant, they are of minimal clinical relevance. No significant age-related changes were evident for the mean ABG for the PTA or for the ABGs at 1000 and 2000 Hz. By contrast with Nondahl et al. [6], we did not find a statistically significant difference between male and female, neither in the whole patient group nor in the younger (20–40 years) or older (60–80 years) groups. 

Since the ossicles could continue development up to approximately 2 years of age, a separate analysis was performed on the 0–10 years group by splitting it into age sub-groups of 0–2 years and 3–10 years. Then, a statistical comparison of the data was done between the two sub-groups, individually for each parameter in Figure 3 and Figure 4. The statistical comparison was based on the same methodology used for the ABG data, described in Section 2.3. For the IMJ, there were 9 and 13 samples for the 0–2 years and 3–10 years age sub-groups, respectively, while for the ISJ there were 7 and 8 samples, respectively. Overall, while there were some trends, there is no statistically significant difference between the two age sub-groups. There was a trend for larger IMJ peripheral (+40% median) and ISJ postline discuss (+24% median) size in 3–10 years versus the 0–2 years age subgroup. In addition, there was a trend for cartilage decrease (up to 20% drop, median) with increased age, in both joints. 

A common and prominent observation was the large variability with age in both the histologic and the audiometric parameters. One may speculate whether some patients experience larger structural changes in the middle ear leading to an increase of the ABG, while others do not. A direct correlation between histologic changes and audiometric test results was obviously not possible in our data, because audiograms of the subjects from our temporal bone collection do not exist. Others correlated histological changes of the middle ear joints to hearing, but only one out of 32 subjects had a conductive hearing loss [18]. Stenklev et al. stated that no change in middle-ear sound transmission occurs with normal aging, as assessed with tympanometry [28]. However, change in middle-ear stiffness with age was suggested by Feeney and Sanford, who found significant age effects in wideband energy reflectance and impedance when comparing young and older adults [20]. Lower stiffness with advanced age were reported by Mazlan et al. examining wideband energy absorbance [29].

Observations from experimental [30] and numerical [31] analyses of the effect of tympanic membrane properties on middle-ear sound transmission may support additional factors in age-related impedance changes. From a mechanical point of view, stiffening the middle ear joints results in better sound transmission at high frequencies, with trends for attenuation at the low frequencies [13]. Widening the joint space of the IMJ, with corresponding softening, should result in the opposite effect, namely, worse sound transmission at high frequencies and potentially better transmission at low frequencies. Such an assumption was supported by modelling of the middle ear [14]. They used a finite element model to investigate the microstructures of the ISJ and found less motion of the footplate with increasing frequency, reaching almost a 6 dB drop at 6 kHz when the joint was less stiff. This change is small and it remains unclear if such a change is clinically relevant, as we also note with respect to our findings. Experimental findings found almost no relative motion between malleus and incus at low frequencies, but increasing relative motion above 3 kHz. Stiffening the IMJ with glue resulted in better sound transmission at high frequencies [29]. The authors argued that the mobility of the IMJ exists as a protection mechanism against static pressure changes. 

The second finding in the histologic analysis was a significant decrease in the cartilage of the incus mainly at the IMJ, but also at the ISJ in the first 20 years of life, while the thickness of the cartilage of the stapes increased with age. Even though the two middle ear joints are fully developed at birth [32,33], the dimensions and mass of the middle ear ossicles increase postnatally [34]. These changes might have an influence on the joint structures, particularly between birth and the age of 20 years. This may be the reason for the thinning of incus cartilage in both middle ear joints. A thinning of the articular cartilage was labelled a Grade II change by Etholm and Belal in their descriptive study [18]. In contrast, we found a constant thickness of hyaline cartilage of the incus and malleus in both joints at older ages. The effect of this change on the ABG cannot be evaluated in our study because only audiograms of subjects older than 20 years of age were analysed, as clinical audiograms and ABGs in particular tend to be less reliable in young subjects. From most clinical experience, there seems to be no change in middle-ear sound transmission in the first 20 years of life due to structural changes, but no clinical studies prove this assumption. No experimental studies shed light into the effect of changing cartilage thickness of the IMJ or ISJ.

One limitation of our study was the use of routine histologic sections of temporal bones with staining every 10th section. Consequently, the section investigated could not have the same plane in all joints, resulting in different measuring points between joints. This may be one reason for the variability of the findings among patients and age groups. Furthermore, the histologic and audiometric data cannot be matched in any direct way because audiometric tests do not exist for the subjects in the temporal bone collection. An attempt was made to reduce the impact of this limitation by including a large number (*n* > 100 for the histologic analysis; *n* > 1000 for the audiometric analysis) of subjects for each of the two vastly different cohorts so that trends could be demonstrated in the statistical evaluation of the data. Finally, although very strict inclusion criteria are applied for the audiometric analysis (i.e., within the adapted ISO-standards (75th percentile)), pathologic ears could have been included in the analysis. The large number of audiograms analysed intends to overcome this possible bias. The two data sets showed small trends that are statistically significant and consistent with modelling of the middle ear. They can be explained from a mechanical point of view; however, the clinical relevance remains unclear.

## 5. Conclusions

The comparison of histological changes of the middle ear joint spaces and the ABGs for 500 Hz, 1000 Hz, 2000 Hz, and 4000 Hz, showed large variability among subjects of the same age group. No general trend could be described at 1000 and 2000 Hz. However, there was a significant decrease at 500 and an increase at 4000 Hz in the ABGs with age. On average, these changes were small and have minimal clinical relevance. However, individual changes at 4000 Hz were large, as much as 20 dB. The widening of the joint space of the IMJ, as observed in the histologic analysis, is a possible explanation for the increase in ABG at 4000 Hz from a mechanical point of view.

## Figures and Tables

**Table 1:** The age (in years, y) and gender distribution of the audiogram data as counts of ears.

Age (Year)	Total (Ears)	Gender		Side	
		Male	Female	Left	Right
20–29	60	38	22	31	29
30–39	343	161	182	177	166
40–49	269	153	116	136	133
50–59	220	124	96	114	106
60–69	476	217	259	244	232
70–80	392	126	266	200	192
**Total**	**1760**	**819**	**941**	**902**	**858**

**Table 2:** Significant histological findings of the IMJ and ISJ.

IMJ				
Parameter	Mean ± SD	Median (Range)	*p*-Value	Age-Related Change
Centerline Discus	89 ± 43	85 (5 to 234)	<0.001	Widening
Peripheral Inc C tot	66 ± 23	61 (28 to 162)	<0.001	Decrease
Peripheral Inc cC	42 ± 19	40 (13 to 128)	0.006	Decrease
Peripheral Inc hC	24 ± 9	22 (9 to 50)	<0.001	Decrease
Peripheral Discus	60 ± 31	58 (4 to 167)	<0.001	Widening
**ISJ**				
Midline Stap C tot	83 ± 29	83 (25 to 166)	<0.001	Increase
Midline Stap cC	59 ± 25	58 (15 to 133)	0.002	Increase
Midline Inc hC	34 ± 12	32 (9 to 77)	0.001	Decrease
Postline Discus	63 ± 43	58 (4 to 223)	0.009	Widening

## Supplementary Materials

The following are available online at https://www.mdpi.com/article/10.3390/jcm10112341/s1, Table S1: Patient characteristics histopathology, Table S2: Statistical analysis IMJ, Table S3: Statistical analysis ISJ.
