# Supplementary material for: Conductive Hearing Loss with Age—A Histologic and Audiometric Evaluation
Source: J Clin Med. 2021 May 27;10(11):2341. doi: 10.3390/jcm10112341 (PMC8198280; doi:10.3390/jcm10112341)
Supplement: Supplementary file 1 [file jcm-10-02341-s001.zip › jcm-1213607-supplementary.pdf]

Supplemental Table S1

	IMJ					ISJ				
		Gender		Side			Gender		Side	
**Age**	**Total**	**Female**	**Male**	**Left**	**Right**	**Total**	**Female**	**Male**	**Left**	**Right**
0-10	22	12	10	11	11	15	9	6	7	8
11-20	15	6	9	3	12	10	6	4	1	9
21-30	23	5	18	5	18	15	1	14	4	11
31-40	21	6	15	7	14	13	5	8	5	8
41-50	21	4	17	10	11	22	4	7	5	6
51-60	25	8	17	10	15	21	9	12	9	12
61-70	26	8	18	5	21	21	7	14	6	15
**Total**	**153**	**49**	**104**	**51**	**102**	**106**	**41**	**65**	**37**	**69**

Supplemental Table S2

IMJ				
**Parameter**	**Mean ± SD**	**Median (Range)**	**p-value**	**Age-related change**
Centerline Discus	*89 ± 43*	*85 (5 to 234)*	*<0.001*	*Widening*
Peripheral Inc C tot	*66 ± 23*	*61 (28 to 162)*	*<0.001*	*Decrease*
Peripheral Inc cC	*42 ± 19*	*40 (13 to 128)*	*0.006*	*Decrease*
Peripheral Inc hC	*24 ± 9*	*22 (9 to 50)*	*<0.001*	*Decrease*
Peripheral Discus	*60 ± 31*	*58 (4 to 167)*	*<0.001*	*Widening*
Centerline B-line	243 ± 67	232 (86 to 475)	0.065	
Centerline Mall C tot	81 ± 41	70 (26 to 255)	0.220	
Centerline Inc C tot	73 ± 28	69 (26 to 161)	0.867	
Centerline Mall cC	52 ± 36	41 (12 to 191)	0.125	
Centerline Inc cC	47 ± 24	42 (11 to 130)	0.748	
Centerline Mall hC	28 ± 14	25 (6 to 76)	0.298	
Centerline Inc hC	26 ± 13	23 (7 to 89)	0.204	
Peripheral B-line	195 ± 48	188 (99 to 342)	0.721	
Peripheral Mall C tot	69 ± 24	63 (29 to 169)	0.019	
Peripheral Mall cC	45 ± 22	41 (15 to 147)	0.084	
Peripheral Mall hC	24 ± 8	23 (8 to 48)	0.012	

Supplemental Table S3

ISJ				
**Parameter**	**Mean ± SD**	**Median (Range)**	**p-value**	**Age-related change**
Midline Stap C tot	*83 ± 29*	*83 (25 to 166)*	*<0.001*	*Increase*
Midline Stap cC	*59 ± 25*	*58 (15 to 133)*	*0.002*	*Increase*
Midline Inc hC	*34 ± 12*	*32 (9 to 77)*	*0.001*	*Decrease*
Postline Discus	*63 ± 43*	*58 (4 to 223)*	*0.009*	*Widening*
Midline B-line	236 ± 60	230 (105 to 374)	0.168	
Midline Inc C tot	101 ± 38	92 (39 to 241)	0.195	
Midline Inc cC	67 ± 38	60 (8 to 206)	0.788	
Midline Stap hC	24 ± 13	23 (5 to 99)	0.182	
Midline Discus	52 ± 29	46 (7 to 134)	0.120	
Antline B-line	263 ± 59	261 (145 to 435)	0.446	
Antline Stap C tot	98 ± 33	97 (26 to 204)	0.082	
Antline Inc C tot	120 ± 39	121 (42 to 220)	0.052	
Antline Stap cC	71 ± 30	69 (13 to 161)	0.060	
Antline Inc cC	85 ± 37	86 (13 to 176)	0.104	
Antline Stap hC	27 ± 14	23 (4 to 68)	0.979	
Antline Inc hC	35 ± 14	33 (12 to 74)	0.266	
Antline Discus	45 ± 31	38 (4 to 137)	0.022	
Postline B-line	280 ± 81	269 (101 to 515)	0.140	
Postline Stap C tot	100 ± 36	97 (33 to 244)	0.166	
Postline Inc C tot	117 ± 45	119 (38 to 296)	0.593	
Postline Stap cC	71 ± 32	69 (8 to 170)	0.068	
Postline Inc cC	84 ± 45	81 (7 to 268)	0.983	
Postline Stap hC	28 ± 14	25 (9 to 96)	0.978	
Postline Inc hC	34 ± 13	33 (3 to 87)	0.018	
